# Supplementary material for: Curated and harmonised transcriptomics datasets of interstitial lung diseases
Source: Data Brief. 2025 Oct 14;63:112139. doi: 10.1016/j.dib.2025.112139 (PMC12581653; doi:10.1016/j.dib.2025.112139)
Supplement: Supplementary file 1 [file mmc1.zip › Supplementary_material/RNA-seq/fastQC reports/GSE184316/SRR15926780_fastqc.html]

SRR15926780.fastq.gz FastQC Report 

FastQC Report

Mon 5 Feb 2024  
SRR15926780.fastq.gz

## Summary

- Basic Statistics
- Per base sequence quality
- Per sequence quality scores
- Per base sequence content
- Per sequence GC content
- Per base N content
- Sequence Length Distribution
- Sequence Duplication Levels
- Overrepresented sequences
- Adapter Content

## Basic Statistics

| Measure | Value |
| --- | --- |
| Filename | SRR15926780.fastq.gz |
| File type | Conventional base calls |
| Encoding | Sanger / Illumina 1.9 |
| Total Sequences | 7435091 |
| Sequences flagged as poor quality | 0 |
| Sequence length | 25-372 |
| %GC | 51 |

## Per base sequence quality

## Per sequence quality scores

## Per base sequence content

## Per sequence GC content

## Per base N content

## Sequence Length Distribution

## Sequence Duplication Levels

## Overrepresented sequences

| Sequence | Count | Percentage | Possible Source |
| --- | --- | --- | --- |
| CAGTGGGGCCACGAGCTGAGTGCGTCCTGTCACTCCACTCCCATGTCCCT | 122063 | 1.6417149433678755 | No Hit |
| AGGCTACACACTGAGGACTCTGTTCCTCCCCTTTCCGCCTAGGGGAAAGT | 108083 | 1.4536876549325355 | No Hit |
| GGGAAGCGGGGAATGTCTACGTGCGTATGCACGTGGCACTCTCTGCCCGA | 84380 | 1.1348885978665224 | No Hit |
| CACCTCCCCGAAGCTCAGGGAGAGCCCTGTTAGGGCCGCCTCTGGCCCTA | 60035 | 0.8074548112457534 | No Hit |
| TGATATGGTTCCTGGCAAGCCCATGTGTGTTGAGAGCTTCTCAGACTATC | 36418 | 0.4898124313475114 | No Hit |
| TTACAAAAGCAGAATAAAAGCGAAAAGAAATGAAAATGTTACACTACATT | 36027 | 0.4845535851545058 | No Hit |
| TGATGACACCCACCGCAACTGTCTGTCTCATATCACGAACAGCAAAGCGA | 34949 | 0.47005477135384083 | No Hit |
| CAATCCACTTGATCCCAACTCATCTCTCATTTATTTCGGCTTCTTTTATT | 34939 | 0.46992027400875114 | No Hit |
| CCCTGGCTGCTACAGGAGAATAGCAGACAGGTATAGTTAAGAAACAACTT | 31602 | 0.4250385099523328 | No Hit |
| AGCTGCTAAGTGCTGTGTTGTCGTTCCCCCTGCTTAAAATAAAGTTGTTT | 29773 | 0.4004389455354346 | No Hit |
| GGCATCAAAAGTGATGGACTGCCCATTGCTGGAGAAGACCTTCTCTCCTA | 28841 | 0.3879037929730786 | No Hit |
| TCAGGAGACGCTCGTAGCCCTCGCGCTTCTCCTCGGCCAATTCGCGGAAG | 26645 | 0.35836817599139 | No Hit |
| TCAGGAGACGCTCGTAGCCCTCGCGCTTCTCCTCGGCCAGTTCGCGGAAG | 25896 | 0.3482943248441747 | No Hit |
| AGACATCAAATCCTGCAGACAAGGGGAGCCCTCAGTCTGCAGGGCTCCAT | 25446 | 0.34224194431514016 | No Hit |
| CAGCAGTTTACACCTGCTAGGGTGTTCAAAGGTCAGTGCTATAGAAATTC | 24733 | 0.3326522836102477 | No Hit |
| AACAAGCACAAAGCCAAGAAAACCAACGATGCCAGATACTGAATTTCTAT | 22616 | 0.30417919565476736 | No Hit |
| TACCACTGCCACCTTCTCCATCGGCTCCACTGGCCTCGTGGTGTATGACT | 20812 | 0.2799158746005933 | No Hit |
| GGCAATGAGCGGTTCCGCTGCCCTGAGGCACTCTTCCAGCCTTCCTTCCT | 20295 | 0.27296236185945805 | No Hit |
| CTTCATGATGTAGCAGCAGGTGCCAGGGGCTGGCTTGTAGGCGATCAGCA | 19123 | 0.2571992730149503 | No Hit |
| GTCCACGTCACACTTCATGATGGAGTTGAAGGTAGTTTCGTGGATGCCAC | 18464 | 0.24833589797354197 | No Hit |
| GAAACCCAGACACATAGCAATTCAGGAAATTTGACTTTCCATTCTCTGCT | 14661 | 0.19718655763594556 | No Hit |
| CAGCATGTTCCCTCTCCTCATGAGATTGGTGAAGAAAGTATTTGGCAAAG | 14205 | 0.1910534786998572 | No Hit |
| CGATCAGCACGGGCTCGTACTCTGCCACAAACTGATCACACTGCTTCTGG | 14056 | 0.18904946825802132 | No Hit |
| CTCTCTTTCTGGCCTGGAGGCTATCCAGCGTACTCCAAAGATTCAGGTTT | 13964 | 0.18781209268319649 | No Hit |
| ACAAGAAGTACATCAAAGATTACATGAAATCAATCAAAGGGAAACTTGAA | 13393 | 0.18013229427857708 | No Hit |
| ACGTTTACCTGTCCATGTCTTACTACTTTGACCGCGATGATGTGGCTTTG | 12901 | 0.173515024900166 | No Hit |
| CAATGGGGTACTTCAGGGTCAGGATGCCACGCTTGCTCTGGGCCTCGTCG | 12331 | 0.16584867623005556 | No Hit |
| GTCTCAACATCTGTGTTGGGGAGAGTGGAGACAGACTGACGCGAGCAGCC | 11710 | 0.1574963910999879 | No Hit |
| CAGGACCACCAATGTCATAGGGTGCAATATCTACAATAGGTAGTCTCACA | 11398 | 0.1533000739331906 | No Hit |
| GGCAATGAGCGGTTCCGGTGTCCGGAGGCGCTGTTCCAGCCTTCCTTCCT | 11013 | 0.14812192614723882 | No Hit |
| GGAGGATGGTTGCACGAAACACACTGGGGAATGGAGCAAAACAGTCTTTG | 10764 | 0.14477294225450638 | No Hit |
| GTCCACGTCACACTTCATGATGGAGTTGAAGGTGGTCTCGTGGATGCCGC | 10714 | 0.1441004555290581 | No Hit |
| GGAGAAAAACAGCACCAAGCAGGAGATCCTGGCTGCTCTTGAGAAAGGCT | 10639 | 0.14309172544088566 | No Hit |
| GTTCTGCAGCCCCTGTCATAAAAGGTTTTACTCTTTCTGGTCTCTGTTCT | 10535 | 0.14169295305195323 | No Hit |
| CGTGAATACCAAGACCTGCTCAATGTTAAGATGGCCCTTGACATTGAGAT | 10149 | 0.13650135553149248 | No Hit |
| CTGACAGTGTATCTAGCTTTGGAAAACACAGGGGTCTGCCCTGTGAGCTG | 9998 | 0.13447044562063867 | No Hit |
| ATCAAAGAAGAACATGTGATCATCCAGGCCGAGTTCTATCTGAATCCTGA | 9939 | 0.1336769112846097 | No Hit |
| CGAAGCGGTTGTACTTGCGGATGTAGTGCAGATAGTCTCGGCGGATGACA | 9243 | 0.1243158960663696 | No Hit |
| GGCAGACAAACCAGACATGGGGGAAATCGCCAGCTTCGATAAGGCCAAGC | 9023 | 0.12135695447439715 | No Hit |
| GGATCTTGGCTTTCTCTTTCCTCTTCTCCTCCAGGGTGGCTGTCACTGCC | 8824 | 0.11868045730711299 | No Hit |
| CCAAGATCTTCTTGATGTCCTTGGCGCTGGGGGAGGAGTTGCCCCCTAGG | 8774 | 0.1180079705816647 | No Hit |
| GCGTCTCTGTCTTCTTCAGTTTCGACTTATCGAATTTCTCGATCTCAGCC | 8601 | 0.11568116651161364 | No Hit |
| CACCAAAGTGCGAAAAGTTACTTTCAATGGACTGAACCAGATGATTGTCA | 8475 | 0.11398649996348398 | No Hit |
| CAGTGGGCCACGAGCTGAGTGCGTCCTGTCACTCCACTCCCATGTCCCTT | 8427 | 0.11334091270705361 | No Hit |
| TCTTGGGTTATAATCATTGGCAATGTTGTAATCAACAACCCATGTTATTT | 8385 | 0.11277602385767706 | No Hit |
| GGAAAGGGGGCACTGACTGGTGATGCTGATATTGGAGTGCATCTGAGGCT | 8312 | 0.11179419323852258 | No Hit |
| TTGGAGTGGGAAGCGGGGAATGTCTACGTGCGTATGCACGTGGCACTCTC | 8193 | 0.11019367483195565 | No Hit |
| GTGTCCATTCGAGGGCGGATCCTCTCTGGCGTGGTGACCAAGATGAAGAT | 8164 | 0.10980363253119566 | No Hit |
| CAGGGAGGAAAAGTTTGGAAGAGGCAGAGAAATCCTGCTCTCCTCGCCTT | 8040 | 0.10813586545208391 | No Hit |
| AGGCTACACACTGAGGACTCTGTTCCTCCCCTTCCGCCTAGGGGAAAGTC | 7475 | 0.10053676545451831 | No Hit |
| CAACTCCTTCATGGTCTCGTCCATCAGCGCCCTCAGTTCCTGGGTGACCT | 7460 | 0.10033501943688382 | No Hit |
| TCGCTCGTACTCGTGCGCCTCGCTTCGCTTTTCCTCCGCAACCATGTCTG | 7452 | 0.1002274215608121 | No Hit |

## Adapter Content

Produced by FastQC (version 0.11.7)
